# Characterizing patient details-related challenges from health information technology-related incident reports from Swedish healthcare

**DOI:** 10.3389/fdgth.2024.1260521

**Published:** 2024-02-06

**Authors:** Md Shafiqur Rahman Jabin, Ding Pan, Evalill Nilsson

**Affiliations:** ^1^ Faculty of Health and Life Sciences, Linnaeus University, Kalmar, Sweden; ^2^ Faculty of Health Studies, University of Bradford, Bradford, United Kingdom

**Keywords:** training and education, system design, patient safety, healthcare quality, quality improvement

## Abstract

**Introduction:**

Despite many benefits offered by Health Information Technology (HIT) systems, new technology brings new and unforeseen risks to healthcare quality and patient safety if they're not properly planned, designed, implemented, and managed. This study examined health information technology-related (HIT) incidents to identify patient details-related issues, their association with contributing factors, and outcomes.

**Methods:**

Sources of information comprised retrospectively collected incident reports (*n* = 95) using two sampling methods, i.e., purposive and snowball sampling. The incident reports were analyzed using both the inductive method (thematic analysis) and the deductive approach using an existing framework, i.e., the International Classification for Patient Safety.

**Results:**

The studies identified 90 incidents with 120 patient details-related issues—categorized as either information-related (48%) or documentation-related (52%) problems; around two-thirds of the 120 issues were characterized by human factors. Of the total sample, 87 contributing factors were identified, of which “medical device/system” (45%) and “documentation” (20%) were the most common contributing factors. Of 90 incidents, more than half (59%) comprised patient-related outcomes—patient inconvenience (47%) and patient harm (12%) and the remaining 41% (*n* = 37) included staff or organization-related outcomes.

**Discussion:**

More than half of the incidents resulted in patient-related outcomes, namely patient inconvenience and patient harm, including disease risks, severe health deterioration, injury, and even patient death. Incidents associated with patient details can cause deleterious effects; therefore, characterizing them should be a routine part of clinical practice to improve the constantly changing healthcare system.

## 1 Introduction

It is evident that interventions related to Health Information Technology (HIT) create viable and timely opportunities to improve accuracy and efficiency in modern medicine (1–3). However, a survey of a nationally representative sample of medical group practices in the US suggested that the adoption of HIT, such as Electronic Health Record (EHR), is slow and complex and requires a great deal of support (4).

For the convenience of the reader, HIT systems comprising e-prescribing and medical imaging systems are used to encompass the sociotechnical systems related to electronic prescriptions and medical imaging (respectively) and the people involved in these systems. The e-prescribing system has been considered a sociotechnical, complex system integrated with other systems, such as Electronic Medical Records (EMRs) with medication lists, National prescription repositories (viewed in mobile or web apps), and pharmacy dispensing systems (5). The components of medical imaging-related HIT systems include the Radiology Information System (RIS), Picture Archiving and Communication System (PACS), etc. (6). Additionally, system integration between the EHR and the patient administration system involving the Care Portal, Care Documentation, and Care Administration system modules/ components has been taken into consideration to indicate various HIT tools involved in this study.

There is ample evidence that patient details can go awry; for example, inflexible electronic forms can result in incorrect orders (7), inaccurate medication requests from a medication ordering system (8), or lost patient data in the EHR system (9). Data entry errors caused by the user can result in incorrect or outdated information remaining in the system and reproducing the same error at several stages of the procedure (10, 11). Healthcare professionals can also delay patient information entry due to their busy schedules or frequent interruptions, resulting in outdated or incomplete information for a longer period (11). “Patient details”, in this context, refer to information-and-documentation-related features of the patient, i.e., any patient information in a healthcare facility is recorded as a document, either in paper or electronic form. For instance, patient demographics and clinical outcomes are usually stored electronically in the health or medical record. Therefore, a record of any patient information has been considered patient details throughout this study.

Several barriers were identified in a recent study by Bjerkan et al. that negatively impacted the nursing practices' documentation process, including individual, social, organizational, and technological factors (12). It was also reported that healthcare professionals found the process of electronic record documentation to be onerous, as such records contain too much information (13). The human factor involves interactions among humans and other elements of a system, optimizing human well-being and overall system achievement (14). On the other hand, the technical factor refers to the attributes of practices and devices/systems that can influence the performance of an organization (15). Although a number of paradigms for improving patient safety have been proposed, the human factors engineering paradigm has been the most prominent for directing improvement efforts. The paradigm focuses on system design to improve the performance of the end users and thus reduce errors, injuries, and hazards (16).

The Swedish Medical Products Agency (MPA) aims to deliver, accord, and contribute to improved healthcare in collaboration with the Swedish eHealth Agency and the Swedish Authority for Privacy Protection. The healthcare providers were recommended to strengthen process measurement and provide leadership to reduce the risks associated with HIT systems (17). Moreover, all Swedish county councils have established computerized reporting systems to which any healthcare practitioner can submit incident reports (18).

According to Magrabi et al., incident reports could be one source among a range of information repositories (18). Reports of HIT-related incidents indicate the gap between the expected and empirically supported HIT advances; therefore, continuous incident reports and analysis could bridge the gap (6, 19–21). An integrated framework for safety, quality, and risk management, including incident management and information systems, was proposed by Runciman et al. (22). The concepts and terms were established as the International Classification for Patient Safety (ICPS)—classification of incident reports and measurement of safety (23). The ICPS helps collect and analyze incident reports to understand what went wrong and how it went wrong.

The incident reports generally contain information regarding the circumstances surrounding the incidents (type of incident), such as what contributed to these events occurring and their outcomes. “Incident type is a descriptive term for a category made up of incidents of a common nature grouped because of shared, agreed features” (23). One incident may be classified into more than one type of issue, for example, information-related issues and documentation-related issues. Information-related issues may reflect on patient information or characteristics, for example, the reason for seeking care, primary diagnosis, and patient status (19, 24). The document-related issues may include any written, typed, drawn, stamped, or printed text or any document where patient information has been entered. Documents may include nursing medical records, protocols or policies, patient labels, stickers, requests, reports, and medical images (19, 24).

According to the ICPS, “contributing factors are the circumstances, actions or influences which are thought to have played a part in the origin or development of an incident or to increase the risk of an incident” (23). Notably, an incident may have more than one contributing factor, and one incident may be a contributing factor to another (a “recursive” model). “Patient outcome relates to the impact upon a patient which is wholly or partially attributable to an incident” (23). On the other hand, “organizational outcomes” refer to the impacts upon an organization which is wholly or partially attributable to an incident” (23). However, readers interested in the conceptual framework, key concepts, terms, and definitions of the “classes” comprising the ICPS may follow a series of articles published by the World Health Organization with the formation of the World Alliance for Patient Safety. Three scientific papers were published in 2009, with the result of the work developed using a two-stage Delphi survey, participated by 300 experts from a range of fields (23, 25, 26).

The thematic analyses and deductive approaches of the ICPS (27) are suitable for analyzing and interpreting HIT incidents in Swedish healthcare. Since little research has been conducted that has focused on issues related to patient details reported in HIT incidents, there is a need for qualitative analysis, both deductive and inductive. This will help explore the challenges related to patient details (information and documentation) that arise in routine clinical practice in Swedish healthcare.

The overall aim of this study was to explore HIT-related incidents and identify patient details-related problems, as well as their association with human and technical factors, using thematic analysis. The study also examines each HIT incident's contributing factors and outcome using the ICPS. This paper explores the following research questions:

1. What patient details-related issues occur in the routine clinical practice of Swedish healthcare?
2. How are these problems associated with human and technical factors?
3. What are the other contributing factors and outcomes of these patient details-related issues?

## 2 Methods

### 2.1 Data collection

Initially, a list of 55 participants was made using purposive sampling, targeting physicians, nurses, medical engineers, and healthcare quality managers covering 21 regions of Sweden. The target population was identified by visiting the official healthcare website of each region and listing each hospital within a region; for example, the contact details for the IT director of Kalmar Region were found on regionkalmar.se. Of the 55 participants contacted, only five responses were received. Due to this low response, an additional 19 participants were approached using snowball sampling, of which 15 responses were collected. The incident reports were collected in two formats depending on the availability and accessibility of the participants. The participants were requested either to participate in interviews (written and telephone) and/or provide a set of retrospectively collected incident reports from their local database.

From the 15 responders, 98 incident reports were collected, ranging from the 5-year period from January 2016 to May 2021. Of the total sample, three were excluded either due to lack of adequate information or inability to categorize it as a HIT incident. The final sample of 95 retrospectively collected HIT incident reports from Swedish healthcare was considered for identifying patient details-related problems and their association with human and technical factors. A detailed description of the participant characteristics from each region, the number of incidents collected, and the time interval of collected incidents are presented in Table 1 (28).

**Table 1:** Characteristics of participants and incident reports.

Participant Characteristics		Region	No. of incidents collected from				Time period of incidents
No.	Occupation		Written response	Telephone interviews	Existing database	Total	
1	Quality manager^1^	Kalmar	1		26	27	01/2016–04/2021
2	Quality Manager^2^	Kronoberg	1		0	1	03/2021
3	Quality Manager^3^	Uppsala	0		38	38	06/2019–06/2021
4	Medical Engineer^1^	Kalmar	1		0	1	03/2021
5	Medical Engineer^2^	Kronoberg	1		0	1	03/2021
6	Medical Engineer^3^	Gävle	0	2	14	16	03/2020–04/2021
7	Medical Engineer^4^	Gävle	0	3	0	3	05/2021
8	Physician^1^	Kalmar	1		0	1	04/2021
9	Physician^2^	Kronobeg	1		0	1	04/2021
10	Physician^3^	Kronobeg	2		0	2	04/2021
11	Physician^4^	Kronobeg	2		0	2	04/2021
12	Physician^5^	Kronobeg	2		0	2	04/2021
13	Physician^6^	Stockholm	1		0	1	04/2021
14	Nurse^1^	Stockholm	1		0	1	03/2021
15	Nurse^2^	Stockholm	1		0	1	04/2021
**Grand total**						**98**	

### 2.2 Data analysis

Incident reports (in the form of free-text narratives) were analyzed using both inductive and deductive methods in order to extract detailed information. The inductive approach involved thematic analysis, proposed by Braun and Clarke (29), whereas the deductive included the ICPS. The thematic analysis was used to determine the incidents with patient details from the total sample (*n* = 95). The thematic analysis involved drawing out a set of keywords or phrases from the total sample that indicated possible relevant concepts. These concepts were then grouped into a number of themes, and the themes of a similar nature were further organized into clusters. Some single themes stood alone and were considered along with the clusters, such as “patient details”. The ICPS was used to identify the contributing factors and outcomes of the incidents. It was shown in the thesis by Jabin et al. that “the contributing factors and outcomes of the ICPS were much more comprehensive than those of the HIT Classification System” (30). Each identified issue was then characterized by either human or technical factors.

Two coders were involved in data analyses (both deductive and inductive) for verification and reliability of the coding. The primary coder performed the thematic analysis, which the secondary coder verified, and the incident was re-examined in case of any disagreement between the coders. An agreement was reached between the coders through dialogue. Interrater reliability using kappa score calculation was performed for the coding of the ICPS, i.e., contributing factors and outcomes, examined by both coders independently. A consensus was reached in case of any difference of opinion.

## 3 Results

Of 95 included incident reports, 77 were from the existing databases of the local hospitals, and 18 were collected through interviews. Of the 18 incident reports collected via interview, 13 were written responses, and five were telephone interviews. All 95 incidents were aggregated for data analysis. The incidents were reported between January 2016 and May 2021. Three major themes emerged from the thematic analysis: HIT incidents affecting multiple patients (28), issues related to HIT system issues (in general), and patient details-related issues. Each theme required separate attention, and the rest of the three themes are beyond the scope of this study.

Of the total sample (*n* = 95), 90 incidents were associated with problems with patient details using thematic analysis, and 120 issues were identified from these problems (see Table 2). Of 90 incidents, 24 incidents comprised two issues, and three incidents resulted in three problems; however, no particular pattern or common theme was found for the incidents with multiple issues. The 90 incidents fell into three main categories: medical records, e-prescribing, and medical imaging. A fourth category, “other,” included clinical chemistry and psychological treatment, which did not fall into the main three categories. When the 90 HIT incidents were allocated to thematic analysis, 120 issues with patient details were identified, more associated with documentation (*n* = 62, 52%) than information (*n* = 58, 48%) (see Table 2).

**Table 2:** Types of issues and their association with human and technical factors.

Types of patient detail issues	HF	TF	*n*	%
Medical record-related				
Information				
Incorrect information	18	0	18	15.00
No/missing/lost information	12	2	14	11.67
Invalid/ irrelevant/ unknown information	6	1	7	5.83
Incomplete information	2	0	2	1.67
**Subtotal (information)**	**38**	**3**	**41**	34.17
Documentation				
No/missing/lost documentation	0	14	14	11.67
Incorrect documentation	9	1	10	8.33
Invalid documentation	6	2	8	6.67
Incomplete documentation	3	0	3	2.50
**Subtotal (documentation)**	**18**	**17**	**35**	29.17
**Total (information + documentation)**	**56**	**20**	**76**	63.33
e-Prescribing-related				
Information				
Incorrect information	9	0	9	7.50
No/missing/lost information	3	0	3	2.50
**Subtotal (information)**	**10**	**0**	**12**	10.00
Documentation				
No/missing/lost documentation	1	4	5	4.17
Invalid documentation	0	4	4	3.33
Incorrect documentation	2	0	2	1.67
Incomplete documentation	0	1	1	0.83
**Subtotal (documentation)**	**3**	**9**	**12**	10.00
**Total (information + documentation)**	**13**	**9**	**24**	20.00
Medical imaging-related				
Information				
No/missing/lost information	1	0	1	0.83
Incorrect information	0	1	1	0.83
Invalid information	1	0	1	0.83
**Subtotal (information)**	**2**	**1**	**3**	2.50
Documentation				
Incorrect documentation	4	1	5	4.17
No/missing/lost documentation	0	4	4	3.33
**Subtotal (documentation)**	**4**	**5**	**9**	7.50
**Total (information + documentation)**	**6**	**6**	**12**	10.00
Other				
Information				
No/missing/lost information	0	2	2	1.67
**Subtotal (information)**	**0**	**2**	**2**	1.67
Documentation				
No/missing/lost documentation	0	2	2	1.67
Delayed documentation	0	2	2	1.67
Incorrect documentation	0	1	1	0.83
Invalid documentation	0	1	1	0.83
**Subtotal (documentation)**	**0**	**6**	**6**	5.00
**Total (information + documentation)**	**0**	**8**	**8**	6.67
**Grand Total (information + documentation)**	**77**	**43**	**120**	

HF, Human factor; TF, Technical factor.

The problems were also classified to check for association with either human or technical factors. Of 120 issues identified, 77 were caused by human factors (64%), and the remaining 43 by technical factors (36%). Within these four groups, each issue was further categorized as either an information-related or documentation-related issue (see Table 2).

Interrater reliability for the outcomes was к (weighted) = 0.89 (*p* < 0·001, 95% CI 0.81–0.98), and for the contributing factors was к (weighted) = 0.87 (*p* < 0.001, 95% CI 0.78–0.96).

NB: It was assumed that all incidents would be associated with patient details; however, five incident descriptions did not contain patient-related information or documentation. For example, the narration of an incident described a service for automatic drug dispensing for which the service unit failed to cut the seam between medicine bags.

### 3.1 Information-related issues

Of the 120 issues, 48% (*n* = 58) comprised information-related problems (see Table 3). An example of an information-related issue may include healthcare professionals omitting patient information or writing inaccuracies in the medical record, such as blood pressure medication.

**Table 3:** Types of information-related issues and their association with human and technical factors.

Types of information-related issues	HF/TF	*n*	%
Medical record-related information			
**Incorrect information**			
Incorrect patient status of care	HF	5	8.62
Incorrect information on diagnosis/treatment/care	HF	5	8.62
Incorrect personal/demographic data	HF	4	6.90
Incorrect information about appointment/booking	HF	3	5.17
Incorrect examination/test data	HF	1	1.72
No/missing/lost information			
No/Missing personal/demographic data	HF	5	8.62
No/missing information about appointment/booking	HF	3	5.17
Missing information about student's health status	HF	2	3.45
No/Missing examination/test data	HF	2	3.45
*No retrieval of personal/ demographic data*	*TF*	2	3.45
Invalid/irrelevant/outdated information			
Invalid information of diagnosis/treatment/care	HF	4	6.90
Irrelevant personal information	HF	1	1.72
*Outdated personal data*	*TF*	1	1.72
Unknown examination data	HF	1	1.72
Incomplete information			
Incomplete clinical history	HF	2	3.45
**Total**		**41**	**70.69**
e-Prescribing-related information			
Incorrect information			
Incorrect dispensing time of medication	HF	4	6.90
Incorrect dose of medication	HF	4	6.90
Incorrect drugs prescribed	HF	1	1.72
**No/missing/lost information**			
Missing information about hypersensitivity (drugs/foods)	HF	3	5.17
**Total**		**12**	**20.69**
Medical imaging-related information			
No/missing/lost information			
Missing concluding information (summary)	HF	1	1.72
Incorrect information			
*Incorrect display of information in medical image*	*TF*	1	1.72
Invalid/irrelevant/outdated information			
Invalid (multiple) tumor iso-centre	HF	1	1.72
**Total**		**3**	**5.17**
Other information			
No/missing/lost information			
*No/missing administrative information*	*TF*	2	3.45
**Total**		**2**	**3.45**
**Grand total**		**58**	** **

HF, human factor (non-italics); TF, technical factor (italics).

Of the 58 patient information-related problems, more than two-thirds involved medical records (*n* = 41; 71%) (see Table 3). Of these 41 issues, 38 were associated with human factors and three with technical factors (see Table 2). For instance, the physician prescribing an incorrect medication was considered to be associated with the human factor, whereas doctors and pharmacists did not have the same view of the medication list attributed to technical factor-related issues. The most common problem with medical records was “incorrect information” (*n* = 18), and all such problems were attributed to human factors.

**Figure 1:**
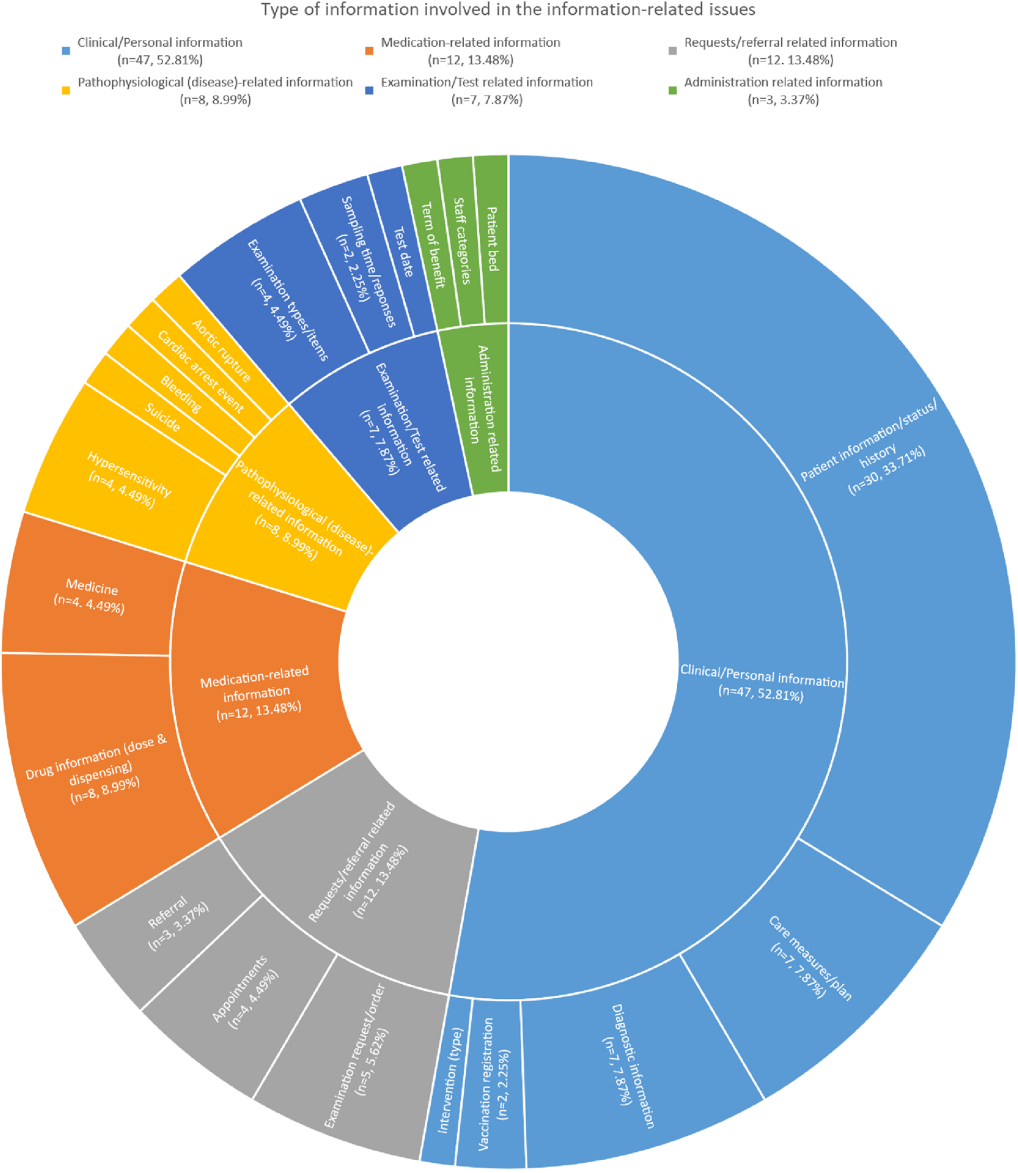
Type of information involved.

A single patient information-related problem may include more than one type of information. For example, incorrect medical record information may encompass several types of demographic or personal information, including name, social security number, or sex. A list of the different types of information is presented in Figure 1, of which more than half comprised clinical/personal information (*n* = 47; 53%).

### 3.2 Documentation-related issues

Of the 120 problems, 52% (*n* = 62) were document-related issues (see Table 4). An illustration of documentation-related issues may involve multiple imaging requests that were required to be written in paper form because of a malfunction of the radiology ordering system.

**Table 4:** Types of documentation-related issues and their association with human and technical factors.

Types of documentation-related issues	HF/TF	*n*	%
Medical records-related documentation			
No/missing/lost documentation			
*Missing patient record*	*TF*	5	8.06
*No/Missing sick/medical certificate*	*TF*	3	4.84
*No/missing request/order/appointment form*	*TF*	3	4.84
*Missing patient report*	*TF*	2	3.23
*Missing waiting list entry*	*TF*	1	1.61
Incorrect documentation			
Incorrect patient record	HF	6	9.68
Incorrect request/order/appointment form	HF	3	4.84
*Another patient's request form*	*TF*	1	1.61
Invalid/outdated documentation			
Unauthorized access/process to medical record	HF	5	8.06
*Outdated documentation*	*TF*	2	3.23
Confused medical record	HF	1	1.61
Incomplete documentation			
Incomplete document for care plan	HF	3	4.84
**Total**		**35**	**56.45**
e-Prescribing-related documentation			
**No/missing/lost documentation**		** **	
*Missing prescription*	*TF*	3	4.84
*No display of "terms of benefit"*	*TF*	1	1.61
No medical prescription sent	HF	1	1.61
**Invalid/outdated documentation**		** **	
*Outdated prescription*	*TF*	2	3.23
*Different drug list*	*TF*	2	3.23
**Incorrect documentation**		** **	
Another patient's prescription	HF	2	3.23
**Incomplete documentation**		** **	
*Incomplete display of prescription*	*TF*	1	1.61
**Total**		**12**	**19.35**
Medical Imaging-related documentation			
Incorrect documentation			
Incorrect medical image obtained/transferred	HF	4	6.45
*Incorrect generation of X-ray referral*	*TF*	1	1.61
No/missing/lost documentation			
*No retrieval of medical image*	*TF*	3	4.84
*No transfer of image*	*TF*	1	1.61
**Total**		**9**	**14.52**
Other documentation			
No/missing/lost documentation			
*No message/chat*	*TF*	2	3.23
Delayed documentation			
*Delayed delivery of the message*	*TF*	2	3.23
Incorrect documentation			
*Incorrect message delivered*	*TF*	1	1.61
Invalid documentation			
*Different alarm logs*	*TF*	1	1.61
**Total**		**6**	**9.68**
**Grand Total**		**62**	

HF, human factor (non-italics); TF, technical factor (italics).

**Figure 2:**
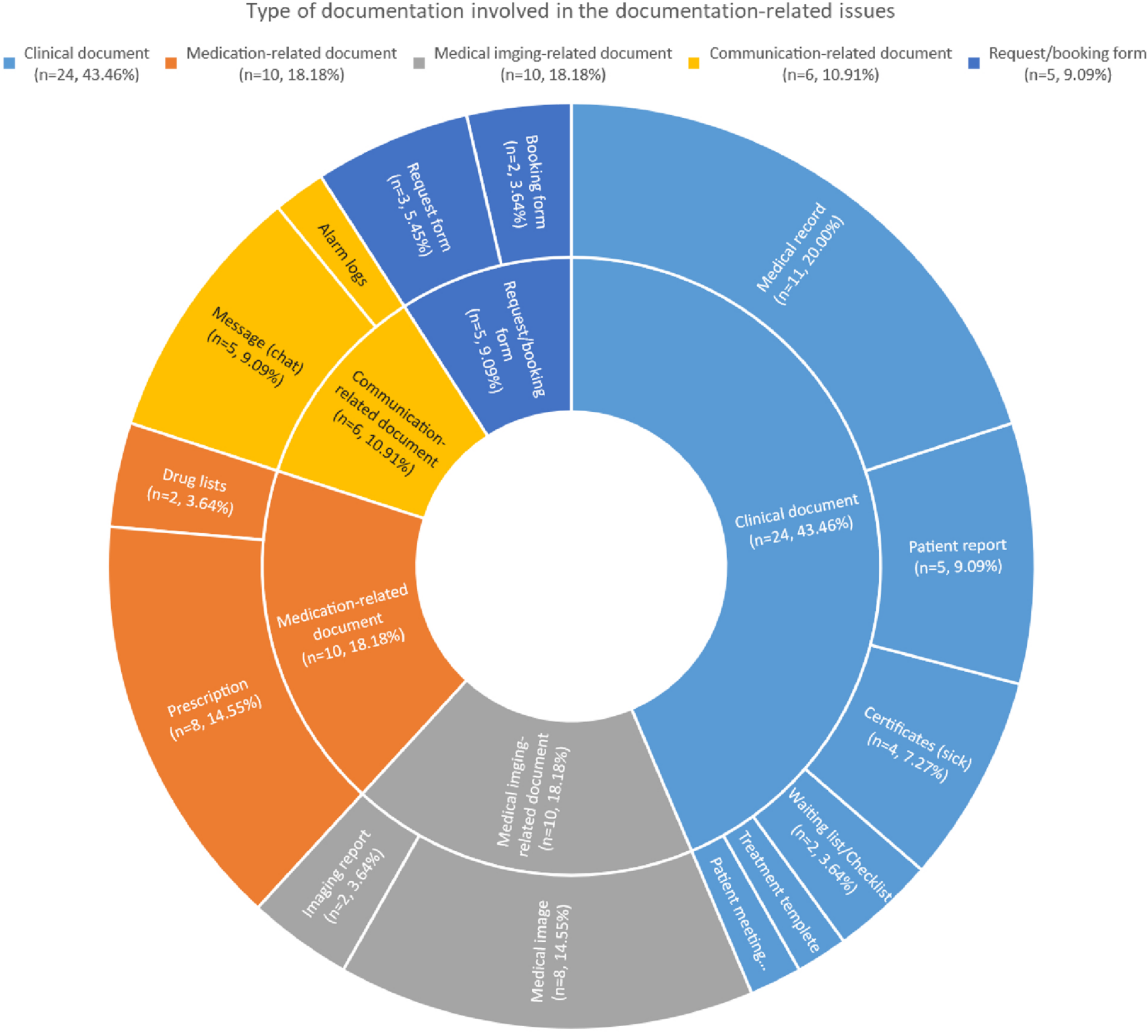
Type of documents involved.

Of the 62 documentation-related problems, more than half were associated with medical records (*n* = 35; 56%) (see Table 4). Of these 35 issues, 18 were attributed to human factors and 17 to technical factors (see Table 2). The most common issue with medical records was “no/lost/missing documentation” (*n* = 14), of which all were associated with technical factors. A list of document types is presented in Figure 2, of which fewer than half comprised clinical documents (*n* = 24; 44%).

### 3.3 Contributing factors

The ICPS was used to capture detailed information about the types of contributing factors associated with those 90 incidents involving patient details-related issues. Of these 90 incidents, 87 contributing factors were identified, of which fewer than half (45%) comprised “medical device/system” factors (*n* = 39) (see Table 5).

**Table 5:** Types of contributing factors involved.

Contributing factors	*n*	%
Medical device/system factor		
Device or system usability	16	18.39
Device or system not working/slow/failed	11	12.64
Device or system suitability for purpose	9	10.34
Device or system unavailable/inaccessible	2	2.30
Device or system unfamiliar	1	1.15
**Subtotal**	**39**	**44.83**
Documentation factor		
Missing/unavailable documentation	7	8.05
Unclear/ambiguous/duplicated documentation	4	4.60
Breach of privacy	4	4.60
Inadequate/incomplete documentation	2	2.30
**Subtotal**	**17**	**19.54**
Staff factor		
Unknown/Not clear	10	11.49
Inattention/distraction/negligence	3	3.45
Knowledge/skills/awareness	3	3.45
Fail to carry out duty	1	1.15
**Subtotal**	**17**	**19.54**
Communication factor		
Not conducted	6	6.90
Inaccurate information communicated	3	3.45
Incomplete	1	1.15
**Subtotal**	**10**	**11.49**
Policy/guideline related factor		
Policy/guideline not followed	2	2.30
**Subtotal**	**2**	**2.30**
Patient factor		
Inattention/distraction/negligence	1	1.15
Knowledge/skills/awareness	1	1.15
**Subtotal**	**2**	**2.30**
**Total**	**87**	

Among medical device/system-related factors, “device/system usability” (*n* = 16) was most common; for instance, the computer system malfunctioned at the beginning of imaging because the imaging modality was turned on in haste. Both documentation (*n* = 17) and staff (*n* = 17) each contributed to one-fifth (20%) of the total factors (see Table 5). Documentation factors were mainly associated with “missing/unavailable documentation” (*n* = 7); for example, a patient summary could not be retrieved because the patient record was missing.

However, staff-related factors were largely unclear or unknown due to insufficient narration of the incident descriptions (see Table 5). In these cases, it was quite clear that healthcare staff contributed to an incident, but information about the type of contributing factor was lacking. For example, a report indicated that staff omitted information or wrote inaccuracies in the medical record, but no indication was given of their reason for doing so.

Regarding “medical device/system,” the most common factor was “device or system usability” (*n* = 16; 18%), indicating that users or healthcare staff had difficulty using the system, which contributed to the incident. For example, a user could not enter complete patient prescription details because benefit terms were not displayed in the intended context. 11% of incidents comprised the communication factor, of which communication was “not conducted” in six cases (see Table 5).

### 3.4 Outcomes

The outcomes of all 90 incidents were identified using the ICPS, and each incident was assigned one outcome. The outcomes were broadly classified into two categories, namely, patient-related, comprising patient inconvenience and patient harm, or staff/organization-related. Of 90 incidents, more than half (59%) comprised patient-related outcomes (*n* = 53)—patient inconvenience (*n* = 42; 47%) and patient harm (*n* = 11; 12%). The remaining 41% (*n* = 37) comprised staff or organization-related outcomes (see Tables 4 and 6).

**Table 6:** Types of outcomes.

Outcomes	*n*	%
Patient-related outcomes		
**Patient inconvenience**	** **	** **
Delays in management diagnosis/procedure/treatment	17	18.89
Unnecessary treatment	9	10.00
Patient dissatisfaction	8	8.89
Repeated or additional diagnosis/procedure/treatment	5	5.56
Financial implications	3	3.33
**Subtotal**	**42**	**46.67**
**Patient harm**	** **	** **
Pathophysiological disease related	6	6.67
Injury	4	4.44
Death	1	1.11
**Subtotal**	**11**	**12.22**
**Total**	**53**	**58.89**
Staff or organization-related outcomes		
More system/service/resource used	14	15.56
Delays in using facilities/service/system	10	11.11
Increased documentation	7	7.78
Phone calls/review/follow-up	6	6.67
**Subtotal**	**37**	**41.11**
**Total**	**37**	**41.11**
**Grand total**	**90**	

Within patient inconvenience, delays in care management (diagnosis/procedure/treatment) accounted for 19% (*n* = 17) of outcomes. Such delays included investigation of the patient treatment plan, care measures, acquisition of images, or medication management. Unnecessary treatment (*n* = 9; 10%) included patient treatment that was not required; for example, patient treatment with blood pressure medication despite any such symptoms or imaging with unnecessary radiation dose. Patient dissatisfaction (*n* = 8; 9%) was expressed either in informal complaints or expressions of dissatisfaction, including decreased confidence in care delivery, suspicions of unauthorized medical records access, or concerns about the competence of the care providers. Repeated or additional diagnosis/procedure/treatment (*n* = 5; 6%) was performed when the healthcare professionals found that the wrong patient was treated/imaged or that the right patient underwent the wrong treatment or examination. These problems caused the same patient to undergo the same imaging, examination, or treatment twice. Three cases of financial implications for patients were identified, including denial of payment or failure to pay compensation or sickness benefits (see Tables 4 and 6).

Six cases resulted in pathophysiological disease-related harm to the patient, including risks of disease or severe deterioration of health due to wrong medication. These comprised four cases of serious injury and one patient death. However, no further information regarding the injury was reported, and the death was not reported to be directly caused by the incident (see Tables 4 and 6).

Of the staff or organization-related outcomes, 16% (*n* = 14) resulted in more equipment/services/resources being used in the form of similar systems or USB sticks, manual extraction/entry of data, or other healthcare professionals, including medical engineers or IT experts. 11% of outcomes resulted in delays in using facilities/service/systems, for example, storage server access delays due to system shutdown; 8% led to increased documentation, such as rewriting imaging orders in paper form; and the remaining 7% resulted in phone calls/review/follow-up, such as contacting other staff involved in the same treatment (see Tables 4 and 6).

## 4 Discussion

For this study, we examined what had gone wrong and how they had gone wrong with HIT systems, including electronic medical records, e-prescribing, and medical imaging, in the daily routine of Swedish healthcare. The exploration of risks to patient details-related safety issues, comprising mainly information and documentation-related problems, established a basis for attempting to determine what steps should be taken to prevent and correct similar problems in the future. Examining what had gone wrong over a period of 5 years, through the lens of 98 incident reports, we expected a host of mainly technical problems.

**Figure 3:**
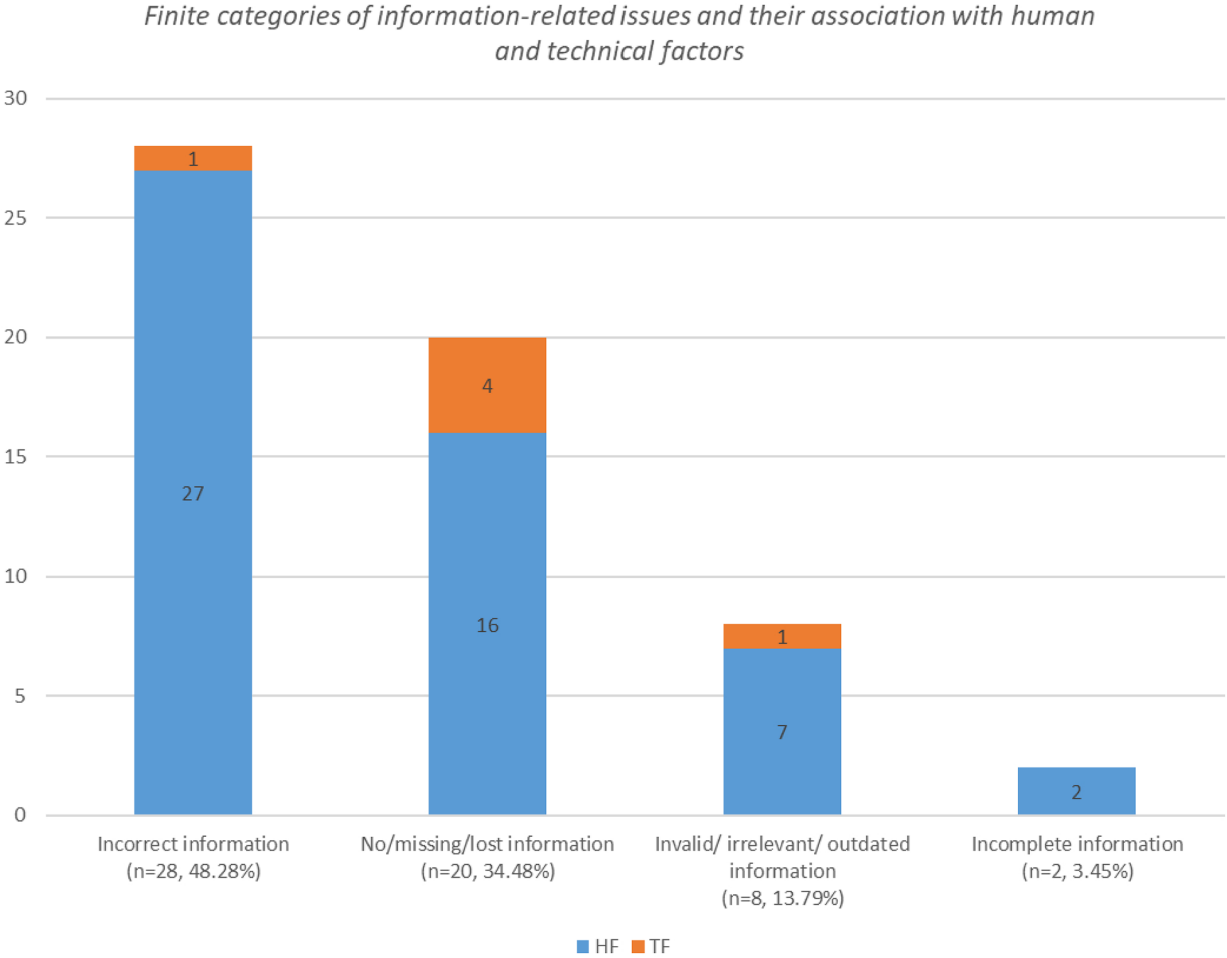
Finite categories of information-related issues and their association with human and technical factors.

Patient details-related issues associated with patient information fell into four finite categories: incorrect information, no/missing/lost information, invalid/irrelevant/outdated information, or incomplete information. There were consistent indications of things going wrong in which human failures played a major role. Among information-related issues, human factors manifested “incorrect information” (27 of 28) and “no/missing/lost information” (16 of 20) (see Figure 3).

**Figure 4:**
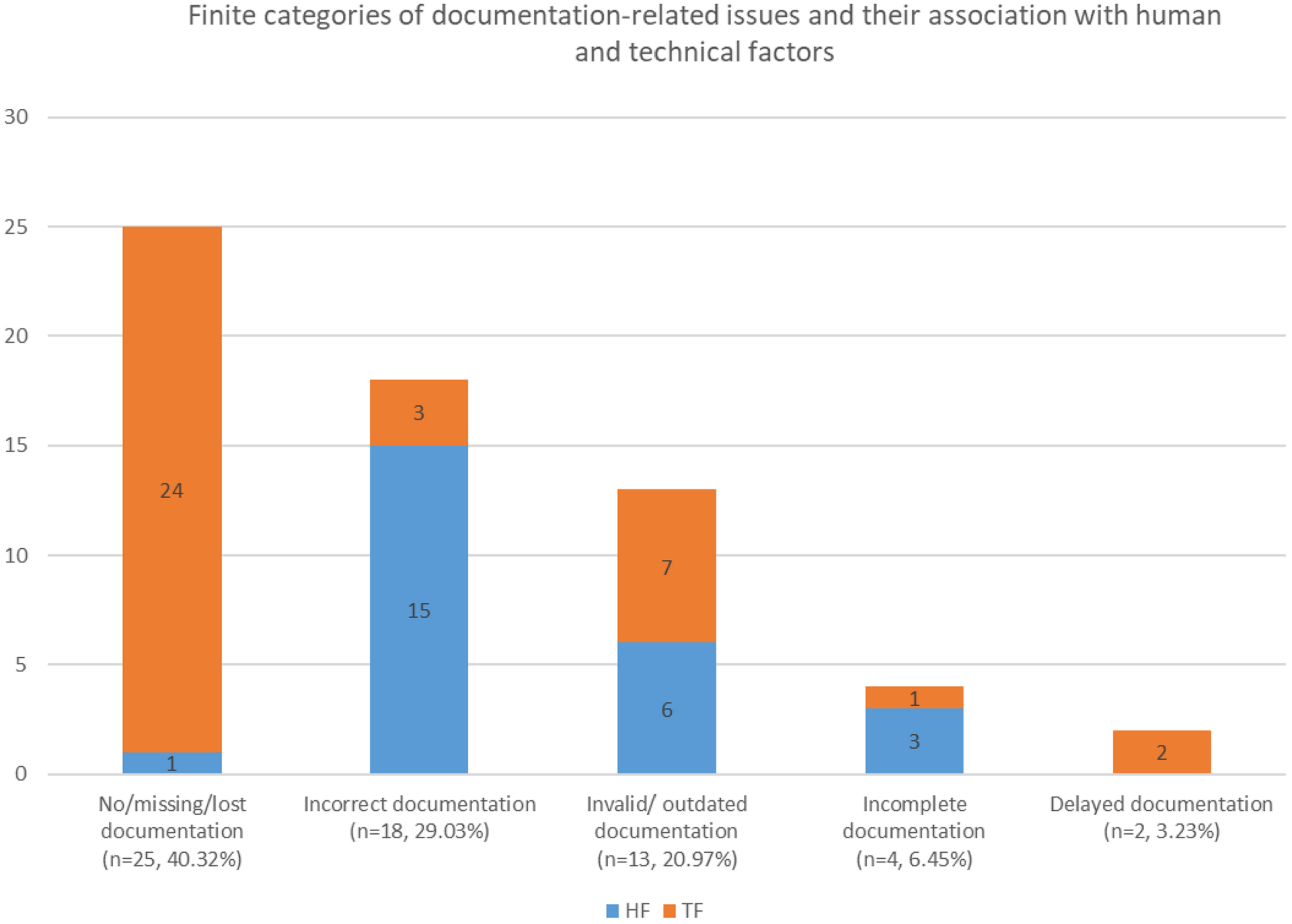
Finite categories of documentation-related issues and their association with human and technical factors.

Documentation-related patient-detail problems fell into five finite categories: no/missing/lost documentation, incorrect documentation, invalid/outdated documentation, incomplete information, or delayed documentation. Among these, human factors predominated in the causation of “incorrect documentation” (15 of 18), while technical factors predominated in “no/missing/lost documentation” (24 of 25) (see Figure 4).

Notably, “medical device/system” and “documentation” were the most common contributing factors, especially when the incidents themselves were HIT system issues (in nature) and involved documentation-related problems (through thematic analysis). This phenomenon confirms the recursive nature of errors and the frequent correlation between types of problems and specific contributing factors. Therefore, the phenomenon may be considered an issue, a contributing factor, or both based on the incident's features or characteristics in that specific context.

### 4.1 Human vs. technical factors

Healthcare is a complex sociotechnical system in which various human factors, including behavior, performance, and culture, play a vital role in building an intimate relationship with the HIT systems. These factors can improve healthcare quality and safety or cause harm and disrupt healthcare processes (31). Even though many technical issues and failures were identified in the reports, we did not expect that more than half of the issues would be caused by human factors (*n* = 77; 64%). However, the incident reports did not contain adequate information to explore further any connection with human behavior, performance, and culture.

Despite the analysis providing no indication of the absolute frequencies of these issues, it does assure the fact that human errors play a vital role in HIT incidents (30). The advantage of systems is that they can be incrementally improved, while the errors of human users are inevitable and remain an inherent part of the complex sociotechnical healthcare system. “Whilst system issues can be progressively ‘designed out,’ it would seem that in the meantime, the rapid availability of experts to diagnose and apply a digital solution to such problems would be highly desirable” (6). Therefore, more considerable thought should be placed on the HIT systems to be designed to prevent specific issues from occurring. This can also be backed up by observational and ethnographic studies that would prevent the occurrence of issues such as those listed throughout this study (30, 32).

### 4.2 Humans as the weak link

The present maturity of the socio-technical HIT system is analogous to that of the early automotive industry. Then, as many as half of all the issues that ended progress during a journey could be ascribed as technical factors and the rest as human factors. At present, “the chance of a technical fault interrupting progress during a journey is minuscule, with virtually all the problems being attributable to human factors on the part of the driver of the vehicle in question, or of another vehicle” (30).

The deployment of some HIT systems has been more successful in some cases than others from the technical point of view, with a special function for automatic detection of malfunction (33)**;** however, the issues of human error still persist, as reflected in this study. A review of 436 HIT incident reports by Jabin et al. reported that human factors are inevitable in the genesis of over 58% of the issues in most complex sociotechnical systems (19). Magrabi et al., in a review of 850 incidents, summarized that human factors were responsible for patient harm four times more often than technical factors (20).

Human error is difficult (if not impossible) to prevent because neither seniority nor experience offers immunity (34). An error occurs through various unintended and unknown cognitive mechanisms beyond human control (35). Even though using the “forcing function” was suggested to prevent such human errors by Norman (35), it would cause “over-proceduralization” that may detract from surveillance and situational awareness. Therefore, it would be suitable to design the system in such a way as to prevent incidents from occurring, as well as provide training to system users on how to best use the system more efficiently (6).

### 4.3 Lack of human-centered design of HIT systems

Even if it is called the “human factor” or “human errors,” it should be clarified that often, it is not individuals who are to be blamed. Rather, it is the complex systems the healthcare professionals work in that are not sufficiently designed (36). Although there was a separation of the human factors from the technical in this study, humans constantly interact with multiple other systems or elements when performing their jobs in complex systems. These include people, job tasks, technology, physical and social environments, the organization of work, and external issues such as regulation and research findings (37, 38). Often, systemic influences and reasons that are either unknown/or unknowable fall under the category of system design–human interaction issues (16, 39).

The concept of “human error” as it applies in complex sociotechnical systems, combined with the lack of human-centered HIT design, causes the system to be unsafe and suffer from usability issues (40). The great majority of data reported, therefore, are, in fact, human factors-related issues. Therefore, blaming the end-user of these systems as contributors to these incidents without extensive evidence occurring in day-to-day clinical practice is not appropriate.

### 4.4 Disruptions in the clinical workflow

In this study, approximately 41% of cases had a staff/organization-related outcome. There was a clear indication that workflow disruptions resulted in additional system/service/resource use and delays in using facilities/service/systems. More than one-third of problems in this study were associated with incorrect patient information or documentation (*n* = 46; 38%). These events caused several risks to patients, such as increased radiation risks, unnecessary procedures, or delays in obtaining correct procedures or medication. Such delays in procedure further delayed diagnosis, treatment initiation, treatment impact monitoring, and decisions regarding future treatment options (continuation, discontinuation, or change in treatment). Once an incorrect piece of information or document is introduced into the system, an “automation bias” tends to be considered it correct (41).

Over the decades, health informatics researchers have been studying the effect of patient information and documentation-related problems that affect the clinical workflow. A review of 149 HIT incidents by Warm and Edwards in 2012 reported that around 34% of the total sample was patient information-related issues, which were categorized into information output (*n* = 25; 16%), information transfer (*n* = 7; 5%), and information input (*n* = 19; 13%) (42). Other studies reported that the quality of the care delivery was compromised by less-than-optimal care or that the risks to patient safety were caused by things going wrong with care delivery (6, 43). Some of the negative impacts include delayed procedures (7), confusion about patient treatments (44), inappropriate decision-making based on incorrect or outdated information (45), and patient harm (40, 44).

### 4.5 Implications for practice

The studies we conducted previously followed a general set of preventive and corrective strategies to improve the healthcare quality and patient safety associated with HIT systems. The strategies recommended are based on the overall challenges encountered by particular HIT systems or types of HIT incidents (5, 28, 46). However, the hallmark of a socio-technical complex system is much more complicated than it seems to be. For example, the manifestation of the issues in this study was evident, and the underlying mechanisms of those incidents were almost obscure. Therefore, we propose strategies associated with the human factors engineering paradigm as a complementary alternative for improving healthcare quality and patient safety instead of the evidence-based risk reduction paradigm (16). More attention has been paid to enabling human-centered system design and a holistic view of healthcare.

#### 4.5.1 The need for training and education programs for healthcare professionals

Healthcare professionals are seldom provided with sufficient training or education for the proper use and operation of HIT systems, manifesting as a lack of proficiency in handling them in healthcare settings (47). For example, a review of 436 HIT incidents by Jabin et al. suggests that system integration and software update-related issues do contribute to human error, causing incorrect entry of patient information and workflow disruptions (6).

Since human error remains an enduring part of the complex healthcare system, it is critically essential to establish an ongoing training process for healthcare professionals in connection with vendors (6). The Joint Commission suggested that healthcare professionals should be adequately trained as significant contributors to HIT-related problems (48). Therefore, training healthcare professionals before integrating HIT systems and software updates and preparing them for unexpected system failures will result in better use of the HIT systems and greater user satisfaction, thus mitigating the risks of human problems (49).

However, professional development to acquire HIT-related skills and competencies can affect patient safety, hindering clinicians from routine practice, such as patient care activities (50). One practical approach to overcoming this barrier is combining conventional classroom and simulation-based training (51). This approach is helpful for novice users but not for experienced participants who may intend to refrain from any additional training (51). Another method is to set aside adequate paid time for balancing the training and their day-to-day clinical practice (6). Basic health literacy programs can also supplement these approaches, which may involve patients in managing their own care (52). For example, a defense mechanism in the general healthcare system can be improved by identifying incorrect prescriptions and providing basic health literacy to patients (53).

Therefore, training healthcare professionals before integrating HIT systems and software updates and preparing them for unexpected system failures will result in better use of the HIT systems and greater user satisfaction, thus mitigating the risks of human problems.

#### 4.5.2 The need for a holistic view of healthcare

A holistic view of the healthcare workflow is necessary to understand and identify the risks. This could include an assessment of risks among various healthcare departments, such as medical imaging, emergency departments, theatres, and Intensive Care Units (ICU). This risk assessment should be accompanied by the ongoing development of new effective strategies to mitigate risk. For example, Jabin et al. reported that failures related to patient information or documentation could occur at any stage, from clinical consultation to clinical action, focusing on the medical imaging workflow process (19). This is true even in our study of the incidents associated with patient identification issues, irrespective of the process workflow in medical imaging or e-prescribing. However, the present study did not consider clinical workflow stages or their association with patient detail issues. Therefore, it is essential to examine different types of patient information and documentation problems, their causation, and their effects on the clinical workflow. This can further help develop the workflow and design solutions addressing particular issues for each healthcare department.

#### 4.5.3 The need for human-centered system design

The core idea of human-centered system design is about understanding human needs and how those needs can be facilitated. To achieve this, Gemma et al. have developed three main characteristics of human-centered system design: understanding people, stakeholder involvement throughout the enter design process, and a system approach toward developing any new products or services (54).

One has to keep in mind that the designers may face some practical challenges, mainly while working in the healthcare context, due to its complex socio-technical nature (54). The practical challenges may include issues associated with conducting fieldwork, managerial difficulties, and resource restrictions, such as time and financial limits. These practical challenges can be resolved in various meaningful ways; for example, effective engagement of stakeholders, careful consideration of vulnerable patient groups, and an adaptation to new and unforeseen restrictions and situations can improve the quality of the fieldwork. The managerial challenges can be overcome through understanding the difference between design and clinical research and continuous engagement of the stakeholders by refining the added value of system design to the stakeholders. The resource allocation can be enhanced by developing an open research environment and creating an effective communication channel among medical specialists, researchers, and stakeholders (55, 56).

Since healthcare organizations are continuously encountering various organizational and societal challenges, they are, therefore, encouraged to adopt an improved form of human-centered system design to facilitate human-centered patient care through multidisciplinary collaboration.

#### 4.5.4 The need for “error-prone features” in system design

Human error can potentially be mitigated by designing systems to prevent incidents from occurring and designing out the “error-prone features”. With considerable thought and ingenuity, the National Health Service (NHS) and the US National Institute of Standards and Technology (NIST) developed and published guidelines and standards for interface design for clinical user interface (57) and EMR usability (58). The HIT industries and national standardization bodies should step in for the design and development of well-established guidelines and standards for safety-critical software. These guidelines and measures should be established based on coveted and suitable working procedures, maintained by national standardization bodies, and backed by government authorities (5).

### 4.6 Strengths and limitations of this study

Incident reports are voluntary, subject to bias, and self-reported. The low response rate was due to the ongoing COVID-19 pandemic, which resulted in a smaller sample than planned. The reporters were not experts in HIT (46), as the reported narrative texts needed to be more accurate, making it impossible to categorize three incidents. For example, an incident was assigned at least one contributing factor; however, it was impossible to classify three incidents to identify even one contributing factor due to insufficient incident description. Therefore, 12% of staff factor-related incidents were categorized as “unknown/unclear” (see Table 5). The inadequacy in the reports also affected the categorization of information-/documentation-related issues; for example, “no” information/documentation was merged with “missing/lost” information/documentation. In addition, most of the issues affected by the “staff factor” could not be identified for the same reason—insufficient narrative texts in the report.

It was a limited data source, and thus it is assumed that incidents were not detected in many cases and not reported even if they were detected (59). Moreover, the total number or frequency of events (failures) can never be compared to successful actions that occur in healthcare. Therefore, the analyses cannot manifest the absolute frequency of the issues, contributing factors, or the identified outcomes. However, the numbers and frequency provide a salient sense of HIT issues that occur in day-to-day clinical practice and demonstrate the harmful role of human error in patient details-related problems.

The incident reports were analyzed using thematic analysis and classified using the ICPS by an expert analyst who previously investigated, analyzed, and classified a large set of incident reports—around 5,000 medical imaging incident reports with a specific focus on over 400 HIT-related medical imaging incidents in Australia (6, 19, 24, 32). The secondary coder was extensively trained to classify the incidents using the ICPS. Also, the ICPS was initially developed without considering the HIT system; therefore, a slight modification of the contributing factor nomenclature “medical device/system” has been considered for this study.

Moreover, combining both deductive and inductive approaches made it possible to extract information that may not be evident using any one analysis method. Moreover, the recursive model of the descriptions of errors and how they occurred constitutes a measure of internal validation for the incident reporting and classification process.

The collection of incident reports ranged over a significant period, validating the feasibility of monitoring incidents regularly. Therefore, new features of the existing problems may have emerged, and further, unforeseen and unprecedented issues may have been identified. The issues identified in this study are compliant with amelioration and mitigation through a systemic approach and rigorous research, such as providing training to healthcare professionals, vigilant system design and implementation, and redesigning the clinical workflow. The issues can be mitigated by permitting the timely application of preventive and corrective strategies at systemic and local levels.

Even though minimal studies on incident analysis have been published, Sweden currently has a comprehensive program on medical device and HIT systems managed by the MPA to ensure quality improvement in Swedish healthcare. To ensure the study generates the generalizable findings, data was collected through various regions, in various formats, such as interviews and retrospectively collected data, and from a range of targeted healthcare professionals across Sweden. Therefore, the results obtained, i.e., the broader categories of the issues, are similar to those previously identified in Australia (30) and the UK (42). This means the lessons learned may be suitable and applicable elsewhere to maintain risk management standards.

## 5 Conclusion

This study provided insight into patient information/documentation-related problems vis-à-vis HIT and how human and technical factors affect patient care delivery. The deductive and inductive approaches analyses provided helpful context to the reporters and analysts regarding where preventive and corrective strategies should be addressed. Therefore, characterizing such HIT incidents and identifying patient details-related problems should be a routine part of clinical practice to improve the constantly changing healthcare system.

## Data Availability

The data analyzed in this study is subject to the following licenses/restrictions: the data are not publicly available since we do not have permission to make part of the data publicly available. Requests to access these datasets should be directed to mdshafiqur.rahmanjabin@lnu.se.
